# Too Good to Keep!

**Published:** 1854-04

**Authors:** 


*Too good to keep!*—A newly appointed Prof. of Theory and Practice in a Western medical school, more familiar with politics than physic, started on a pilgrimage to the east. At Buffalo he called upon one of the magnates of the profession. In the course of the conversation the *nouveau ne* Prof. inquired as follows: "Can you tell me, Sir, what there is about this matter of physical diagnosis? Is it really, now, worth knowing?"

The host indicated very politely that it was, perhaps, desirable that *teachers* should have some knowledge of it; whereupon the newly elected one said "that if it was really worth while, he would go down to New York for a fortnight, and acquire it. For his part he had n't much faith in it."

Whether or no our ambitious teacher followed the advice of his host, "to go by all means," we cannot say.

